# Alignment of microbial fitness with engineered product formation: obligatory coupling between acetate production and photoautotrophic growth

**DOI:** 10.1186/s13068-018-1037-8

**Published:** 2018-02-13

**Authors:** Wei Du, Joeri A. Jongbloets, Coco van Boxtel, Hugo Pineda Hernández, David Lips, Brett G. Oliver, Klaas J. Hellingwerf, Filipe Branco dos Santos

**Affiliations:** 1 Molecular Microbial Physiology Group, Faculty of Science, Swammerdam Institute for Life Sciences, University of Amsterdam, Science Park 904, 1098 XH Amsterdam, The Netherlands; 2 Systems Bioinformatics/Amsterdam Institute for Molecules, Medicines and Systems (AIMMS)/Netherlands Institute for Systems Biology, VU University Amsterdam, De Boelelaan 1085, 1081 HV Amsterdam, The Netherlands; 3 Modelling of Biological Process, BioQuant, Heidelberg University, Im Neuenheimer Feld 267, 69120 Heidelberg, Germany

**Keywords:** Strain stability, Growth coupled, Metabolic modeling, Cyanobacteria, Acetate production

## Abstract

**Background:**

Microbial bioengineering has the potential to become a key contributor to the future development of human society by providing sustainable, novel, and cost-effective production pipelines. However, the sustained productivity of genetically engineered strains is often a challenge, as spontaneous non-producing mutants tend to grow faster and take over the population. Novel strategies to prevent this issue of strain instability are urgently needed.

**Results:**

In this study, we propose a novel strategy applicable to all microbial production systems for which a genome-scale metabolic model is available that aligns the production of native metabolites to the formation of biomass. Based on well-established constraint-based analysis techniques such as OptKnock and FVA, we developed an *in silico* pipeline—FRUITS—that specifically ‘Finds Reactions Usable in Tapping Side-products’. It analyses a metabolic network to identify compounds produced in anabolism that are suitable to be coupled to growth by deletion of their re-utilization pathway(s), and computes their respective biomass and product formation rates. When applied to *Synechocystis* sp. PCC6803, a model cyanobacterium explored for sustainable bioproduction, a total of nine target metabolites were identified. We tested our approach for one of these compounds, acetate, which is used in a wide range of industrial applications. The model-guided engineered strain shows an obligatory coupling between acetate production and photoautotrophic growth as predicted. Furthermore, the stability of acetate productivity in this strain was confirmed by performing prolonged turbidostat cultivations.

**Conclusions:**

This work demonstrates a novel approach to stabilize the production of target compounds in cyanobacteria that culminated in the first report of a photoautotrophic growth-coupled cell factory. The method developed is generic and can easily be extended to any other modeled microbial production system.

**Electronic supplementary material:**

The online version of this article (10.1186/s13068-018-1037-8) contains supplementary material, which is available to authorized users.

## Background

**Fig. 1:**
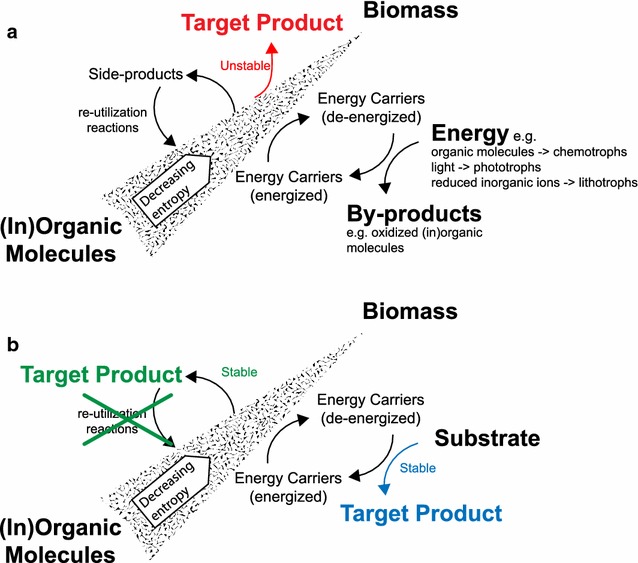
Schematic representation of the main mass and energy balances involved in biomass formation, along with alternative metabolic engineering strategies to make target compounds. **a** Target product formation is metabolically engineered using classical approaches that redirect native fluxes toward it. Product formation and biomass formation compete for carbon and energy, which eventually leads to strain instability. **b** Overview of growth-coupled metabolic engineering strategies. Others have proposed to tie fluxes to biomass and target product by linking energy and/or redox regeneration to product formation (blue). We propose here to achieve this by knocking out the genes encoding pathways responsible for re-utilizing the side products of anabolism (green)

The advent of genetic engineering in the 1970s imbued us with the ability to rationally tailor microorganisms to our needs [1]. Although immediately recognized for its biotechnological potential [2], four-and-a-half decades later, the usage of genetically engineered organisms in large-scale industrial processes still faces some significant technical hurdles [3]. An important example of such challenges is the process unpredictability that emerges from the instability of engineered strains in production settings [4]. This occurs because conventional metabolic engineering strategies often cause high fitness trade-offs for production strains, as product is made in direct competition with biomass formation (Fig. 1a). This ultimately leads to a rapid appearance of suppressor mutations, for instance in the form of insertions or deletions, which impair the culture’s ability to form the product [5, 6].

Cyanobacteria that can use (sun) light as their sole energy source can be genetically engineered to directly convert CO_2_ and water into compounds of interest [7]. This is highly relevant for its potential to mitigate climate change and thus contribute to the increased sustainability of society. However, also here the emergence of suppressor mutations strongly compromises the economic viability of such processes, making them difficult to scale-up without significant productivity losses [8]. In laboratory experiments, engineered strains have been reported to be unstable for the products ethylene [9], lactate [10], isopropanol [11] and mannitol [12]. Given that negative results are often ignored in science, there have likely been many more occurrences of genetic instability in practice, to the point that the genetic instability problem in cyanobacteria has been called an “*elephant in the room*,” as it is “*important, obvious*, *yet largely ignored*” [13]. Recently, we have directly demonstrated that the fitness burden of product synthesis is associated with deviating carbon itself from biomass to product formation [14]. This unsettling observation suggests that a certain degree of culture instability is apparently inevitable using the genetic engineering approaches implemented for photoautotrophs thus far.

Enforcing obligate coupling of bacterial growth with the synthesis of specific target products—commonly termed *growth*-*coupled production*—can help stabilize production traits. By aligning microbial fitness with product formation, Darwinian selection ensures that spontaneously occurring non-producing mutants are outcompeted by the fitter producing strains from the growing population [15]. One can find organisms for which this concept has evolved in nature for a limited range of products. As an example, many fermentative chemoheterotrophs secrete products, such as butanol, acetone and ethanol, in a growth-coupled fashion to maintain a closed redox balance in the cell [16].

Significant efforts have been made, both *in silico* and in vivo, to engineer growth-coupled production in microbes. For instance, in *Escherichia coli* there are several successful cases of growth-coupled product formation being reported for lactate [17], 1-butanol [18], succinate and malate [19] and *iso*-butanol [20]. However, a recent screen based on the analysis of its genome-scale metabolic model (GSM) concluded that many more products can potentially be produced using this approach [21]. Remarkably, the latter authors found that the production of all metabolites present in central metabolism could in principle be obligatorily coupled to growth under aerobic conditions. Although no experimental evidence has been provided for this claim so far, this suggests that stable product formation may be engineered for several compounds.

The idea of growth-coupled production has also been proposed and discussed in the literature for cyanobacteria [22–26], but has never been implemented experimentally. Similar to what has been done for chemoheterotrophs, the theoretical framework applied to cyanobacteria involved coupling a product-forming pathway to the ability of a metabolic network to regenerate energy and/or redox co-factors. This has been achieved in GSM simulations by closing or limiting the *in silico* flux through any pathway that is responsible for it in the wild-type organism (Fig. 1b), while simultaneously ensuring that the flux through the proposed production pathway is the only feasible alternative to produce energy and/or balance the redox potential. In *Synechocystis* sp. PCC6803 (hereafter, *Synechocystis*), a very detailed analysis has been carried out for the production of reduced solvents (e.g., ethanol), finding that at least five genes associated with alternative electron flow need to be blocked to ensure growth coupling [25]. Even with the latest CRISPR-based mutagenesis technology [27, 28], the attempt to test such predictions will still require significant time and effort, without guaranteed in vivo results. More importantly, deletion of alternative electron flow pathways would undoubtedly lead to a strain that is more fragile under varying light conditions [29] and hence be very difficult to cultivate in industrial settings.

Here, we present a strategy to align microbial fitness with product formation that is, in principle, applicable to any microbial production system. Rather than using energy or redox regeneration, we instead propose to directly (i.e., stoichiometrically and obligatorily) couple the production of target compounds to pathways uniquely responsible for the formation of biomass precursors. This can be achieved by deleting the native metabolic routes that cells have to reintroduce side products formed in anabolic pathways. This will lead to their accumulation and ensure growth-coupled production (Fig. 1b).

We have developed a new algorithm to analyze GSMs that allows identification of target products, along with the necessary modifications to the network to guarantee that their formation is growth coupled. We tested this approach on a published metabolic network reconstruction available for *Synechocystis*, and found *in silico* that the synthesis of at least nine products could be stoichiometrically coupled to the formation of biomass precursors. This approach was then experimentally validated for one of these predicted products, acetate, to showcase its feasibility. We have thus achieved the first growth-coupled cyanobacterial cell factory, using an approach that can be applied to any organism for which a GSM is available.

## Methods

### *In silico* simulation and tools

The genome-scale stoichiometric model *i*JN678 [30] was used to simulate light-limited photoautotrophic growth of *Synechocystis*. The model topology and its numerical parameters (e.g., reaction stoichiometry, biomass equation composition, and lower and upper bounds of metabolic fluxes) were left unchanged. With these default settings, using the maximization of biomass formation as the objective function, the selected lower bound of the photon uptake flux (30 µmol photons m^−2^ s^−1^) limits the biomass synthesis rate (0.052 gDW h^−1^). The newly developed algorithm was implemented as a single script within a Python package containing the complete analysis pipeline (https://gitlab.com/mmp-uva/fruits). All constraint-based analysis techniques used within this pipeline, namely flux balance analysis (FBA) [31], flux variability analysis (FVA) [32] and OptKnock [33], were performed using PySCeS-CBMPy [34] (http://cbmpy.sourceforge.net) in combination with ILOG CPLEX Optimization Studio (IBM) under an academic license. Additional visualization of modeling simulations were carried out using a resource particularly developed for *Synechocystis* [35] available at the FAME online modeling environment [36].

### Strains and general cultivation conditions

All molecular cloning procedures were carried out in *E. coli* DH5α, either grown on solidified LB plates, containing 1.5% (w/v) agar, or in liquid LB medium at 37 °C in an incubator with a shaking speed of 200 rpm. When appropriate, antibiotics were added to the medium for propagation of specific plasmids. Concentrations of antibiotics used, alone or in combination, were 100 µg mL^−1^ for ampicillin and 50 µg mL^−1^ for kanamycin.

A glucose-tolerant *Synechocystis* derivative was obtained from D. Bhaya, University of Stanford, Stanford, CA. Normally, it was cultivated in BG11 medium [37] at 30 °C in a shaking incubator at 120 rpm (Innova 43, New Brunswick Scientific) under constant moderate white light illumination, ~ 30 μmol photons m^−2^ s^−1^, measured with an LI-250 light meter (LI-COR, Inc). For *Synechocystis* mutant construction, kanamycin or nickel sulfate was added to the medium with a final concentration of 50 µg mL^−1^ or 20 µM, respectively. Biomass concentration in the cultures was monitored by recording the optical density at 730 nm (OD_730_) in a spectrophotometer (Lightwave II, Biochrom).

### Plasmid and *Synechocystis* mutant engineering

All strains and plasmids are listed in Table 1. To construct the markerless gene knockout strains, a counter-selection approach was used [38]. This approach uses two plasmids to markerlessly delete a locus from the *Synechocystis* chromosome. First one selection plasmid, containing the up- and downstream homologous regions (~ 1 kb each) of the target locus, and a selection cassette in between, is used to replace the native sequence. Then, another plasmid with only the up- and downstream homologous regions is used to remove the selection cassette. The selection cassette consists of kanamycin resistance and nickel-induced *mazF* expression fragments [38]. MazF acts as a general inhibitor for the synthesis of all cellular proteins by displaying endoribonuclease activity that cleaves mRNA at the ACA triplet sequence [39]. To construct the required plasmids, first the corresponding sets of the homologous regions of *acs* and *ackA* were amplified from the genomic DNA of *Synechocystis*, and separately fused together using *Pfu* DNA Polymerase (Thermo Scientific). After gel extraction and purification (Zymo Research), an extra adenosine (“A”) was attached to the 3′ end of each fused fragment using *Taq* DNA Polymerase (Thermo Scientific). TA cloning enables ligation of these fragments to the BioBrick “T” vector pFL-SN [40], resulting in pWD001 and pWD003, respectively. To obtain the selection plasmid, either an *Xba*I or *Avr*II restriction site was introduced between the homologous regions through designed fusion primers for, respectively, pWD001 and pWD003. Since the selection cassette (from pWD42) contains an *Xba*I restriction site on both sides, it could easily be inserted into pWD001 and pWD003, resulting in pWD002 and pWD004, respectively. All the fragments amplified in this study were confirmed by Sanger sequencing at Macrogen Europe (The Netherlands), and all the primers used are listed in Additional file 1: Table S1.

**Table 1:** Plasmids and strains used in this study

Plasmids and strains	Relevant characteristics	References
pFL-SN	BioBrick “T” vector with SpeI and NheI on each side	[40]
pWD42	Amp^r^Km^r^, containing the selection cassette	[58]
pWD001	pFL-SN derivate, Amp^r^, containing *acs* gene upstream and downstream homologous regions	This study
pWD002	pFL-SN derivate, Amp^r^Km^r^, containing *acs* gene upstream homologous region, selection cassette and downstream homologous region	This study
pWD003	pFL-SN derivate, Amp^r^, containing *ack*A gene upstream and downstream homologous regions	This study
pWD004	pFL-SN derivate, Amp^r^Km^r^, containing *ack*A gene upstream homologous region, selection cassette and downstream homologous region	This study
*Synechocystis* sp. PCC6803	*Synechocystis* sp. PCC6803 wild type	D. Bhaya
WD023	*Synechocystis* sp. PCC6803 *acs* gene knock out mutant	This study
WD025	*Synechocystis* sp. PCC6803 *ack*A gene knock out mutant	This study
WD027	*Synechocystis* sp. PCC6803 *acs* and *ack*A double gene knock out mutant	This study

*Synechocystis* mutant construction takes two rounds of transformation to achieve a markerless gene deletion. The first round is to fully integrate the selection cassette into the chromosome through homologous recombination, while the second round is to completely remove it again. To transform *Synechocystis* with the plasmids, fresh cells were collected either directly from the plate or from liquid culture (OD_730_ ≈ 1). After being washed twice with fresh BG11 medium through centrifugation (5000 rpm, 5 min), cells were further concentrated to a total volume of 200 µL (OD_730_ ≈ 2). The desired plasmid was mixed with these cells to a concentration of 10 μg mL^−1^, and then the mixture was illuminated with white light of moderate intensity (~ 50 μmol photons m^−2^ s^−1^) for 4–6 h. Next, the mixture was spread on a commercial membrane (Pall Corporation), resting on a BG11 agar plate (without marker selection). After further illumination for about 16–24 h, the membrane containing the mixture of cells was transferred to a new BG11 plate, supplemented with kanamycin (first round) or nickel sulfate (second round). After about 1 week, colonies were picked and streaked sequentially on a new BG11 plate with kanamycin and a plate with nickel sulfate (first round), or a new BG11 plate with nickel sulfate and a plate with kanamycin (second round). Colonies which grew on the BG11 plate with kanamycin but not on plate with nickel sulfate (first round), or on a BG11 plate with nickel sulfate but not on a plate with kanamycin (second round), were candidates for PCR confirmation. Further segregation by serial dilution in liquid culture under the same conditions was applied when necessary.

### Batch cultivation

Batch cultivations were carried out in the Multi-Cultivator (MC1000-OD, PSI, Czech Republic), with emitted light intensity controlled through a LED panel, equipped with “cool-white” LEDs (PSI, CZ). All experiments were performed at 30 °C in BG11 medium supplemented with 10 mM TES–NaOH (pH = 8), and bubbled with a mix (v/v) of 99% N_2_ and 1% CO_2_ at a flow rate of ~ 150 mL min^−1^. Pre-cultures from shake flasks were used for inoculation of cultures with a 60 mL working volume (area exposed to the light = 0.0028 m^2^, height of the water column = 10.32 cm) in the multi-cultivator, at an initial OD_730_ of ~ 0.05. Fixed light intensity of 30 µmol photons m^−2^ s^−1^ (calibrated at the back of the vessel filled with BG11 medium) was given after inoculation and was increased to 120 µmol photons m^−2^ s^−1^ at OD_730_ ≥ 0.5. Samples were collected daily for both external OD_730_ determination and for extracellular broth analysis.

### Photonfluxostat

Photonfluxostat is a method for light-limited cyanobacterial batch cultivation at different, yet constant, growth rates [41]. It is based on the dosage of light proportionally to the cell density such that the emitted light per unit of biomass is constant. Performed in a Multi-Cultivator, with its built-in OD sensor, the photonfluxostat illumination regime is implemented by an open-source in-house software package (https://gitlab.com/mmp-uva/pycultivator), which supervises online OD_720_ measurements at regular intervals. Operation conditions adopted for the photonfluxostat were similar to the ones described above for the batch cultivations except for the light intensity settings. Briefly, light intensity was also initially set to 30 µmol photons m^−2^ s^−1^ immediately after inoculation. But when OD_720_ reached 0.6, the light intensity was automatically adjusted every 5 min to ensure that the light intensity per OD_720_ was constant. This light regime was then maintained until the maximum capacity of the LED panel was reached (~ 300 µmol photons m^−2^ s^−1^, as measured at the back of the vessel filled with BG11 medium). During photonfluxostat cultivation, we grew 12 independent cultures, at different growth rates, via illumination at different light intensities per biomass concentration [specifically, 30 (twice), 40, 45 (twice), 50, 60 (twice), 70, 80, 90, 100 µmol photons m^−2^ s^−1^ OD^−1^]. Supernatant samples were collected at regular intervals for analysis of the acetate concentration.

### Turbidostat cultivation

We studied the genetic stability of our strains in populations maintained under turbidostat mode [42]. In this continuous cultivation method, microbial populations are kept at a fixed biomass density by diluting the culture with fresh medium at the same rate as the populations grows. This feedback loop applies a strong selection pressure on cells to grow at the maximal specific growth rate [43]. The turbidostat setup used in this experiment is based on a modified Multi-Cultivator, with additional pumps (Reglo ICC, ISMATEC, Germany) transferring fresh medium to the cultures and, subsequently, to a waste container (i.e., as in a classical chemostat). The “pycultivator” software package that controls the Multi-Cultivator and adjunct hardware additionally sets the pumps to dilute the cultures if the selected OD_720_ threshold is reached. Cells from pre-cultures in shake flasks were inoculated at OD_720_ ~ 0.05 in four independent cylindrical vessels of the Multi-Cultivator, using the same conditions as specified before, except for the incident light intensity, which was fixed at 50 µmol photons m^−2^ s^−1^. The OD_720_ was recorded every 5 min. When the threshold of OD_720_ > 0.35 was reached, cultures were diluted by 8% (v/v) with fresh BG11. Strain stability was assessed by monitoring the growth rate and acetate production in time. The growth rate was calculated by fitting a linear function through the natural logarithm of the OD_720_ during each cell “growth–dilution” cycle. Samples for acetate production were collected periodically throughout the cultivation period. The variation in growth rate, expressed in percentage, was calculated relative to the one observed at the beginning of the cultivation experiment. A similar approach was used to analyze the variability in acetate productivity.

### Assay of acetate concentration in spent medium

Extracellular acetate concentration was determined in samples collected from cultures of the different strains under different conditions using a commercial enzymatic acetate assay (Megazyme). Cells were removed from 0.5 mL culture sample by centrifugation for 5 min at 13,000 rpm at 4 °C. Supernatant samples were stored at − 20 °C for later use. For the enzymatic acetate assay, approximately 200 µL of a supernatant was used according to the manufacturer’s instructions. During the assay, the conversion of the acetate present in the sample to acetyl phosphate is stoichiometrically coupled to the conversion of NADH to NAD+. The latter conversion was quantitated via the absorption (340 nm) of the sample, which was performed in 96-well plates (Greiner), using a plate reader (BMG FLUOstar OPTIMA Microplate Reader), operated at room temperature. For quantification, the assay was calibrated with a standard curve obtained under the same conditions.

## Theory and *in silico* analysis

### Obligate coupling of product formation to the flux through anabolic pathways

Several reactions within anabolic pathways have side products, besides the intermediates that are synthesized to eventually be incorporated in various biomass components. Cells tend to be equipped with metabolic routes to reintroduce these side products back into metabolism, as this generally enables a more efficient use of resources (Fig. 1a). This recycling is particularly important when these side products are related to burdensome cellular processes such as carbon fixation by photoautotrophic organisms. This may also partly explain why organisms like *Synechocystis* tend to have an extended anabolic versatility underlying their very limited number of auxotrophies [44].

The strategy to achieve growth-coupled production explored in this study relies on the disruption of the endogenous reactions that are responsible for the recycling of the side products, which are stoichiometrically associated with the synthesis of biomass precursors (e.g., amino acids, co-factors, lipids). The growth of such mutant strains obviously still requires the production of the biomass precursors and, consequently, formation of the side products. But since the latter cannot be reintroduced into metabolism (as the enzyme(s) carrying this out were disrupted), these very specific compounds will accumulate (Fig. 1b). This may happen intracellularly and/or in the culture, depending on the presence of a transport system, on the thermodynamic drive, and/or on the tendency of the particular compound to leak across the membrane. Either way, in such strains, growth-coupled production is ensured as the synthesis of a side product is tightly coupled to that of an essential biomass precursor.

### Introducing “FRUITS”

We have developed an algorithm to ‘Find Reactions Usable in Tapping Side-products’—FRUITS (Additional file 1: Figure S1). It analyzes existing GSMs and enables the identification of side products of anabolism that are suitable to be coupled to growth of the cells by deletion of their respective re-utilization pathway(s) (Fig. 1b). A GSM of the desired production organism, suitably constrained (e.g., mimicking industrial conditions), is the only required input. Applying the pipeline, implemented as a series of Python scripts, FRUITS produces (i) a list of all the modeled metabolites that can be produced in a growth-coupled manner, (ii) the gene deletions that are required to do so, and (iii) the computed maximal biomass formation rate along with the predicted minimum flux toward the identified target compounds.

FRUITS is initiated by evaluating the input GSM for reactions responsible for the synthesis of macromolecules (e.g., proteins, DNA or RNA) and their precursors (e.g., amino acids or nucleotides). All identified reactions are then dissected into their required substrates and products. A distinction for the products is drawn between biomass precursor metabolites (i.e., strictly used within the anabolic pathway) and co-produced molecules (i.e., that are not necessarily directly used as a substrate in an anabolic pathway). The list of co-produced compounds is then trimmed by removing molecules that do not contain carbon (e.g., Mg^2+^, NH_4_^+^ or PO_4_^3−^) or that are co-factors that act as energy carriers or redox equivalents (e.g., ATP, NADPH, NADH or CoA). The remaining metabolites constitute the list of preliminary growth-coupled candidates that will be considered in subsequent steps of the pipeline.

To ensure that compound excretion can be predicted using a GSM, it is required that it can be exchanged within the boundaries of the defined system. In modeling terminology, this means that a metabolite must be a *boundary species* for which a so-called *sink reaction* is present. The metabolites in the preliminary list of candidates do not necessarily have a sink reaction, which would not allow them to ever be predicted to accumulate. This is resolved by creating a model version for each compound of the list in which a single new sink reaction is added, if necessary, for the respective target compound. These multiple models with the appropriate sink reaction present are then further analyzed individually.

The possibility of changing the topology of the metabolic network of each of these models, such that the respective candidate compound is made in a strict growth-coupled way, is then evaluated. In essence, we want to find whether there are gene deletions that completely disrupt the re-utilization of a target compound, and if so, which one(s). This is achieved using an in-house Python implementation of OptKnock [33], extended for gene deletions [45]. The user can choose the maximum number of permitted deletions such that it satisfies experimental limitations. OptKnock is very useful to identify the gene deletions necessary to maximize the flux toward a target compound; however, it does not guarantee that the product is uniquely coupled to an anabolic pathway. The strict stoichiometric coupling between the rate of formation of biomass and product are therefore tested by additionally performing FVA on the constrained model with the proposed gene deletions, while using the maximization of biomass formation as the objective function. If the minimum flux rate through the exchange reaction is greater than zero, then the possibility of making this metabolite growth coupled is confirmed (at least *in silico*!).

Ultimately, this algorithm will report a list of all the identified target compounds along with the necessary associated gene knockouts and the maximal biomass and minimum target product formation rates. This information can then be used directly to guide the engineering of stable producers, and/or be combined with alternative metabolic engineering strategies (e.g., heterologous expression) that may further boost productivity.

## Results and discussion

### Analysis of the GSM of *Synechocystis* using FRUITS

We tested the newly developed FRUITS using *i*JN678—a published genome-scale metabolic model of *Synechocystis*—with default constraints that support a light-limited growth rate of 0.052 h^−1^ [30, 35]. The first step of the pipeline of identifying side products resulted in a list of 80 preliminary candidates (Additional file 1: Table S2) out of a total of 817 metabolites present in the model. Next, while allowing a maximum of four gene deletions, we found nine target metabolites (Table 2) as the output of FRUITS. The predicted product yield on biomass (*Y*_p/x_) of each target metabolite was calculated based on its production rate and the maximal growth rate. In this particular case, for all the nine metabolites, FVA showed that the minimum and maximum flux rates predicted were identical, suggesting indeed a very strict stoichiometric coupling—just as intended.

**Table 2:** Metabolites that can be produced in a growth-coupled way based on *in silico* simulations

Metabolite	Growth rate (h^−1^)	Yield (mmol gDW^−1^)	Gene knockouts
5-Methylthioadenosine	0.052	0.007	sll0135
Acetate	0.052	0.195	sll0542, sll1299
Mercaptopyruvate	0.034	5.702	sll1027 or sll1502, slr0710, sll1499
5′-Deoxyadenosine	0.052	0.044	sll1185
3,4-Dihydroxy-2-butanone 4-phosphate	0.051	0.732	sll0753, sll0330, sll1556
Adenine	0.052	0.032	sll1430
Adenosine	0.052	0.032	sll1430
S-Adenosyl-l-homocysteine	0.052	0.025	sll1758
Fumarate	0.051	0.848	slr0018
	0.044	3.162	slr0018, slr0458, sll1349
	0.043	3.509	slr0018, slr0458, sll1349, slr1755

**Fig. 2:**
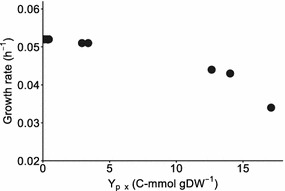
Relationship between predicted growth rate and carbon product yield on biomass for each of the compounds listed by FRUITS to be coupleable to growth in *Synechocystis*. Each point represents an individual compound (and a mutant strain with dedicated gene deletions as indicated in Table 2)

The output of FRUITS, when applied to *i*JN678, reveals some interesting patterns. For instance, the higher the maximum number of allowed gene deletions, not only the greater is the number of target products that are identified, but also the greater are the predicted production rates (see the case of fumarate in Table 2). Here, we have limited this parameter to four, in pace with the common ability to genetically engineer these strains in the laboratory [46]. Another observed pattern is the inverse proportionality between the predicted maximal growth rates and the production rates normalized over the number of carbon atoms per molecule (C-mol)—i.e., the more the carbon is deviated toward product formation, the slower will the culture grow (Fig. 2). This *in silico* prediction is consistent with a recent study in which the same effect was observed for lactate production [14]. There, a clear drop in growth rate was observed when the flux toward lactate was allosterically increased without changing the expression level of the responsible enzyme (l-lactate dehydrogenase).

The list of target compounds identified by FRUITS mostly contains entries for which a strategy to achieve growth-coupled production has not been proposed yet. To the best of our knowledge, the only exception is fumarate, which had been previously singled out [23]. However, it is important to note that FRUITS has here revealed additional gene deletions not considered in the previous report, which are predicted to lead to even higher production rates of fumarate. Many of the compounds in the list are of biotechnological interest, covering a variety of potential applications. For instance, adenosine and adenine can be used for pharmaceutical and nutritional applications [47], respectively, while fumarate and acetate have been listed as relevant for the biobased economy [48, 49]. The fact that all of them can now in principle be produced directly from CO_2_ in a sustainable and stable fashion may be of great value.

We experimentally validated the feasibility of this approach for one of the compounds in the list—acetate—by introducing the required genetic modifications and monitoring their effects on biomass and product formation rates. Acetate has a large market size (e.g., 13 million tons in 2015) reflecting its multiple industrial applications, ranging from food additive to chemical intermediate ingredient [48]. In biotechnological processes, it may serve as an accessible carbon source that has been shown to be easily converted into compounds such as poly-3-hydroxybutyrate [50, 51] and isopropanol [11]. Additionally, acetate is often adopted as a cross-fed carbon source in co-culture systems [52, 53].

### Acetate metabolism in *Synechocystis*

**Fig. 3:**
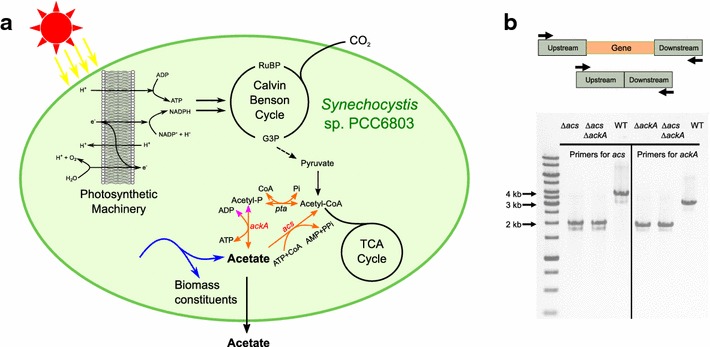
Acetate metabolism and mutant construction in *Synechocystis.* **a** Schematic representation of acetate producing (blue) and consuming (orange) pathways, revealed by the genome-scale model (GSM) [30]. Acetate is also produced as an anabolic by-product and secreted (by an as of yet unknown mechanism). Genes (red and italic) related to acetate consuming pathways were deleted in this study. Pink arrowheads indicate the direction of the AckA predicted in the GSM, but found to be infeasible under the experimental conditions tested. **b** PCR confirmation of the strains constructed for markerless deletion of *acs* and *ackA*. With the primers on each side of the upstream and downstream homologous region (~ 1 kb each), a markerless construct gave a PCR product ~ 2 kb, while the wild-type band was about 4 and 3.2 kb for *acs* and *ackA*, respectively

According to the model *i*JN678 [30], under light-limited conditions, acetate is produced as a by-product of four independent anabolic pathways (Fig. 3a): (i) the reaction catalyzed by *O*-acetyl-l-homoserine acetate-lyase that converts *O*-acetyl-l-homoserine to l-homocysteine, an intermediate of methionine synthesis; (ii) in the synthesis of cysteine, through which cysteine and acetate are generated from acetyl serine and hydrogen sulfide, catalyzed by cysteine synthase (encoded by *sll0712* or *slr1842*); (iii) during the step catalyzed by UDP-3-*O*-acetylglucosamine deacetylase (encoded by *sll1508*) for lipid A synthesis; and (iv) in the route of biosynthesis of vitamin B_12_, during the step catalyzed by precorrin-6A synthase. The flux through each of these pathways is predicted by FBA to be responsible for 50, 23.9, 25.9 and 0.2%, respectively, of the total flux toward acetate. It is important to clarify that under these conditions, none of these predicted fluxes toward acetate directly taps from central carbon metabolism or from appending fermentative pathways. This is further supported by experimental data on acetate formation during diel L/D regimes (12 h light/12 h dark) [54]. There, during the light period, it is clearly shown that acetate is not formed from glycogen via central carbon metabolism.

The flux through all these four anabolic pathways is obviously required for balanced growth. Since light-limited wild-type *Synechocystis* cultures do not accumulate acetate, this implies that it must be internally re-assimilated. Given the energetic burden that CO_2_ fixation carries, it is not at all surprising that these cells have evolved metabolic routes to conserve the energy that has been invested in formation of C–C bonds. As described in *i*JN678 [30], there are two alternative routes to channel acetate back into the metabolic network hub acetyl–CoA (Fig. 3a). One, directly via the activity of acetyl–CoA synthetase (Acs) encoded by *sll0542* that irreversibly converts acetate, CoA and ATP into acetyl–CoA, AMP and pyrophosphate (PPi). Another, via a two-step pathway composed of the (erroneously predicted—evidence provided below) reversible reaction catalyzed by the acetate kinase (AckA) encoded by *sll1299*, plus the reversible activity of a phosphate acetyltransferase (Pta), encoded by *slr2132*. The overall stoichiometry of the latter pathway differs from the one catalyzed by Acs only in that ADP and Pi are formed rather than AMP and PPi. According to the output of FRUITS (Table 2), if both of these acetate re-utilization routes would be disrupted, then, and only then, would acetate accumulate in the culture in a growth-coupled way. More precisely, a *Y*_p/x_ of 0.19 mM gDW^−1^ is predicted.

### Acetate production in wild-type *Synechocystis* and derivative strains

**Fig. 4:**
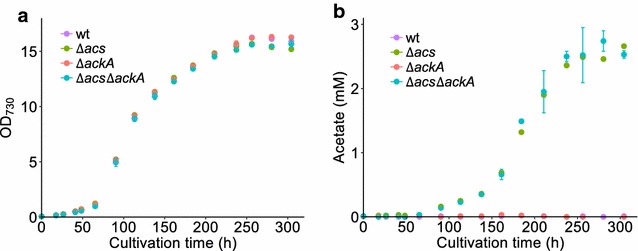
Acetate production in different *Synechocystis* strains. Growth curves (**a**) and acetate concentration (**b**) in different strains from Multi-Cultivator experiments with continuous illumination. Error bars represent the standard deviation of at least two biological replicates. If an error bar is not visible, it is smaller than the point symbol

We tested these *in silico* predictions by constructing, both alone and in combination, markerless deletion mutants of *acs* (*sll0542*) and *ackA* (*sll1299*), using an established protocol (Fig. 3b) [38]. Then we cultured wild-type *Synechocystis* and the *∆acs*, *∆ackA*, and *∆acs∆ackA* mutants in a controlled batch photocultivation in a Multi-Cultivator under constant illumination for 300 h. During these cultivations, the biomass and acetate concentrations were monitored daily. The growth curves of the four strains were very similar (Fig. 4a), as predicted by the model (Table 3). With respect to acetate accumulation (Fig. 4b), the wild type and the single *∆ackA* mutant did not produce detectable amounts, while the single *∆acs* and the double *∆acs∆ackA* mutants secreted large amounts of acetate reaching a titer of > 2.5 mM after 300 h. The acetate concentrations measured during photoautotrophic growth of the wild-type, *∆ackA* and *∆acs∆ackA* strains were as anticipated (Table 3); however, for *∆acs* they were not. In fact, the mutant carrying a single deletion of *acs* produces the same levels of acetate as the *acs* and *ackA* double mutant. This suggests two things: (i) the acetate formed is indeed not a product of the fermentative AckA–Pta pathway under these conditions with continuous illumination; and (ii) AckA does not play a role under these conditions in the re-utilization of acetate, which implies that the activity of Acs alone is responsible for this. This second conclusion is in direct conflict with the model prediction, and so we decided to explore it further both theoretically and experimentally.

**Table 3:** Predicted and experimentally observed growth rate and product yield of acetate on biomass in wild-type *Synechocystis* and derivative strains impaired in acetate metabolism

Strains	Growth rate (*µ*, h^−1^)		Acetate yield (*Y*_p/x_, mM gDW^−1^)	
	Model prediction	Measured	Model prediction	Measured^a^
Wild type	0.052	0.048 ± 0.002	0	0
*Δacs*	0.052	0.049 ± 0.001	0	0.201 ± 0.011
*ΔackA*	0.052	0.048 ± 0.001	0	0
*Δacs ackA*	0.052	0.047 ± 0.002	0.192	0.193 ± 0.007

^a^Measured yield was calculated based on data collected during the exponential cell growth phase (65–120 h) presented in Fig. 4b. Dry weight concentration was extrapolated from the OD_730_ measurements using a conversion coefficient determined in this same setup of 148 mg L^−1^ OD_730_^−1^ [41]

### Acs serves as the main metabolic acetate assimilation pathway

We started by analyzing the reaction catalyzed by AckA, which converts acetate and ATP to acetyl-P and ADP, using a biochemical thermodynamics calculator, eQuilibrator [55]. We then estimated ∆*G* as a function of the Log_10_ concentration ratios of ADP to ATP and acetyl-P to acetate, rather than the more commonly used products over substrates, because of the scarcity of data on absolute intracellular metabolite concentrations available for *Synechocystis*. We calculated that, even considering the highest ATP:ADP ratios reported [56, 57], the concentration of acetate has to be at least ~ 100-fold larger than that of acetyl-P for the net flux through the reaction to be positive in the forward direction, i.e., ∆*G* < 0 (Additional file 1: Figure S2). This ratio is highly unlikely under the conditions simulated, and therefore it strongly suggests that the constraints of the AckA reaction (R_ACKr) in the model be revised such that it would be defined as irreversible. When this is done, model predictions and experimental observations are aligned.

**Fig. 5:**
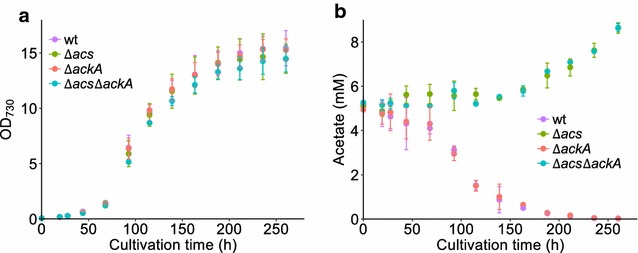
Acetate consumption/production in wild-type *Synechocystis* and derivative strains impaired in acetate metabolism while cultured in BG11 medium supplemented with 5 mM acetate. **a**, **b** Biomass content and acetate concentration, respectively, in Multi-Cultivator batch experiments under constant illumination. Error bars represent the standard deviation of at least two biological replicates. If an error bar is not visible, it is smaller than the point symbol

We did, however, decide to perform one additional experiment in which pressure would be applied on the thermodynamic drive of AckA in the reverse direction. This was achieved by supplementing batch cultures of *Synechocystis* and derivative strains, growing in a Multi-Cultivator, with an exogenous supply of acetate (5 mM) and monitoring daily the concentrations of both biomass and acetate (Fig. 5a, b). The acetate initially added to the cultures of both wild type and the *∆ackA* mutant was completely consumed throughout the cultivation, at similar rates for both strains. In contrast, for the *∆acs* and *∆acs∆ackA* mutants, the 5 mM acetate initially added appears to not only have remained untouched, but also the extracellular concentration of acetate kept increasing as the cultures became denser.

The evidence provided by these two approaches convinced us that only Acs is required for acetate assimilation. However, the prediction that acetate production in this *∆acs* mutant is coupled to growth still remains to be tested directly.

### Acetate production coupled to photoautotrophic growth

**Fig. 6:**
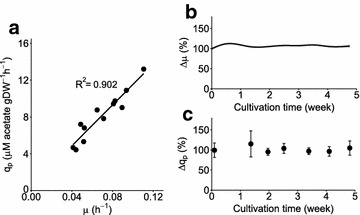
Stable growth-coupled acetate production in a *Synechocystis ∆acs* mutant. Photonfluxostat cultures at different light intensities per biomass concentration reveal the proportionality between biomass-specific acetate productivity and growth rate (**a**). Steady states maintained in four independent turbidostat replicates display constant growth rate (**b**) and biomass-specific acetate productivity (**c**) for over 1 month of cultivation. Dry weight in **a** was calculated based on OD_730_ measurements using a conversion coefficient of 148 mg L^−1^ OD_730_^−1^ previously determined for this setup [41]. Each filled circle in **a** represents a single observation, while gray shaded area in **b** and error bars in **c** indicate the standard deviation from four biological replicates

The relationship between the rate of acetate production (*q*_p_, mM acetate gDW^−1^ h^−1^) and the growth rate (*µ*, h^−1^) for the *∆acs* mutant was explored under constant light-limited conditions during steady-state exponential growth. To achieve this, we applied the new mode of cultivation, the photonfluxostat, by dosing the incident light flux according to the biomass density [41]. This method allows for the light-limited batch photocultivation of photoautotrophs at different, yet constant, growth rates, in which we simultaneously determined the extracellular acetate concentration. We observed a good linear fit (*R*^2^ = 0.902) for the relationship between *µ* and *q*_p_ (Fig. 6a). This indicates that the acetate production rate is proportional to the growth rate and, therefore, provides direct evidence that for this strain, acetate is indeed produced in a strictly growth-coupled fashion.

The metabolic engineering strategy applied here is expected to lead to stable production. This is because the timescale required to evolve an enzyme capable of re-utilizing acetate is likely many orders of magnitude larger than the longest biotechnological process one could conceive of. We nonetheless decided to test whether growth-coupled production in fact leads to robust and sustained product formation. For this purpose, four independent biological replicate cultures of the *∆acs* mutant were tested in prolonged turbidostat experiments, in which there is a strong selection pressure on growth rate [43]. During the cultivation, we monitored online the OD_720_ from which the growth rate was inferred (Fig. 6b), and periodic samples were taken to determine the rate of acetate production (Fig. 6c). Over the entire course of the cultivation (> 1 month), we could not see a statistically significant difference for both the biomass and product formation rates. One could question whether the cultivation time for this experiment was sufficiently long. To answer this it is useful to note how rapidly non-producing mutants can be observed for other production strains in which biomass and product formation rates are not stoichiometrically coupled [14]. Based on this, we argue that if the *∆acs* mutant would be genetically unstable, in these cultivations there would have been sufficient time to see clear signs of it. We therefore interpret these results as a validation that growth-coupled production indeed has the potential to stabilize the productivity of a target compound, even when the latter is produced at high rates.

## Conclusions

In this study, based on a combination of modeling and experimental approaches, we have been able to engineer the first photoautotrophic growth-coupled cell factory. Its design is based on an algorithm that is general and applicable to any organism for which a GSM is available. The alignment of microbial fitness with product formation is a solution that directly tackles issues of strain instability, which are a major hurdle in the scale-up of many biotechnological processes. The *in silico* pipeline presented here, along with the proof of principle provided in the form of stable photoautotrophic growth-coupled production, will hopefully elicit wider application of this promising metabolic engineering strategy.

## Additional file

**Additional file 1.** Additional figures and tables.
