# Supplementary material for: Alignment of microbial fitness with engineered product formation: obligatory coupling between acetate production and photoautotrophic growth
Source: Biotechnol Biofuels. 2018 Feb 13;11:38. doi: 10.1186/s13068-018-1037-8 (PMC5809919; doi:10.1186/s13068-018-1037-8)
Supplement: Supplementary file 1 — Additional file 1. Additional figures and tables. [file 13068_2018_1037_MOESM1_ESM.pdf]

Additional File 1

Title

Alignment of microbial fitness with engineered product formation: Obligatory coupling between acetate production and photoautotrophic growth

Authors

Wei Du[1], Joeri A. Jongbloets[1], Coco van Boxtel[2], Hugo Pineda Hernández[1], David Lips[1], Brett G. Oliver[2,3], Klaas J. Hellingwerf[1] and Filipe Branco dos Santos[1,*]

Author affiliation

[1] Molecular Microbial Physiology Group, Faculty of Sciences, Swammerdam Institute for Life Sciences, University of Amsterdam, Science Park 904, Amsterdam 1098 XH, The Netherlands

[2] Systems Bioinformatics/Amsterdam Institute for Molecules, Medicines and Systems (AIMMS)/Netherlands Institute for Systems Biology, VU University Amsterdam, De Boelelaan 1085, 1081 HV Amsterdam, The Netherlands

[3] Modelling of Biological Process, BioQuant, Heidelberg University, Im Neuenheimer Feld 267, 69120, Heidelberg, Germany

***Corresponding author**

Filipe Branco dos Santos

f.brancodossantos@uva.nl

Additional Tables

Table S1 Primers used in this study

Names	Primers (5’ to 3’)
ackA-upst-Fwd	CACAACGGTGGTTACCTTTAAGTAACAACAGTTACAATTTGTGAT
ackA-upst-Rev	CATTTTCGCAATTTACGcctaggTCATTTCAATTCGGTTTGGA
ackA-downst-Fwd	TCCAAACCGAATTGAAATGAcctaggCGTAAATTGCGAAAATG
ackA-downst-Rev	CCATTCCCCTGGTGGGCTTACTGTTCATTG
acs-upst-Fwd	TTCCACTTCACTTGGTTTGTCAACAGTGCCACC
acs-upst-Rev	TCCAACACGCTATTAGtctagaGACATGGTTCTCCGTCAAAG
acs-downst-Fwd	CTTTGACGGAGAACCATGTCtctagaCTAATAGCGTGTTGGA
acs-downst-Rev	CCCACGCCGATCCTGATTTATATCCTGACCCAG

Table S2 Identified candidate metabolites during step 1 of FRUITS when analyzing the genome-scale metabolic model of *Synechocystis i*JN678

Species Id	Species names
M_dcamp_c	N6-(1,2-Dicarboxyethyl)-AMP
M_fpram_c	2-(Formamido)-N1-(5-phospho-D-ribosyl)acetamidine
M_5mta_c	5-Methylthioadenosine
M_hisp_c	L-Histidinol phosphate
M_csn_c	Cytosine
M_4hpro_LT_c	trans-4-Hydroxy-L-proline
M_cgly_c	Cys-Gly
M_ump_c	UMP
M_ac_c	Acetate
M_2mecdp_c	2-C-methyl-D-erythritol 2,4-cyclodiphosphate
M_4h2oglt_c	4-Hydroxy-2-oxoglutarate
M_mercppyr_c	Mercaptopyruvate
M_ncam_c	Nicotinamide
M_4adcho_c	4-amino-4-deoxychorismate
M_4mop_c	4-Methyl-2-oxopentanoate
M_lac_D_c	D-Lactate
M_pre6a_c	Precorrin 6A
M_acald_c	Acetaldehyde
M_aicar_c	5-Amino-1-(5-Phospho-D-ribosyl)imidazole-4-carboxamide
M_pre5_c	Precorrin 5
M_4abz_c	4-Aminobenzoate
M_dhap_c	Dihydroxyacetone phosphate

M_pyr_c	Pyruvate
M_fprica_c	5-Formamido-1-(5-phospho-D-ribosyl)imidazole-4-carboxamide
M_4ppcys_c	N-((R)-4-Phosphopantothenoyl)-L-cysteine
M_acglu_c	N-Acetyl-L-glutamate
M_3mop_c	(S)-3-Methyl-2-oxopentanoate
M_pre3a_c	Precorrin 3 A
M_glu5sa_c	L-Glutamate 5-semialdehyde
M_2dhp_c	2-Dehydropantoate
M_sl2a6o_c	N-Succinyl-2-L-amino-6-oxoheptanedioate
M_anth_c	Anthranilate
M_cmp_c	CMP
M_dad_5_c	5'-Deoxyadenosine
M_suc6p_c	Sucrose 6-phosphate
M_db4p_c	3,4-dihydroxy-2-butanone 4-phosphate
M_ade_c	Adenine
M_sucsal_c	Succinic semialdehyde
M_hgbam_c	Hydrogenobyrinate a,c diamide
M_pram_c	5-Phospho-beta-D-ribosylamine
M_ahdt_c	2-Amino-4-hydroxy-6-(erythro-1,2,3-trihydroxypropyl)dihydropteridine triphosphate
M_4r5au_c	4-(1-D-Ribitylamino)-5-aminouracil
M_g3p_c	Glyceraldehyde 3-phosphate
M_udp_c	UDP
M_2shchc_c	2-Succinyl-6-hydroxy-2,4-cyclohexadiene-1-carboxylate
M_pre4_c	Precorrin 4
M_5g2oxpt_c	5-Guanidino-2-oxopentanoate
M_dna5mtc_c	DNA 5-methylcytosine
M_dscl_c	dihydrosirohydrochlorin
M_acmana_c	N-Acetyl-D-mannosamine
M_oaa_c	Oxaloacetate
M_adcobhex_c	Adenosyl cobyrinate hexaamide, Adenosylcobyric acid
M_pppg9_c	Protoporphyrinogen IX
M_mppp9om_c	Magnesium protoporphyrin monomethyl ester
M_34hpp_c	3-(4-Hydroxyphenyl)pyruvate
M_26dap_LL_c	LL-2,6-Diaminoheptanedioate
M_g1p_c	D-Glucose 1-phosphate
M_phpyr_c	Phenylpyruvate
M_gmp_c	GMP
M_akg_c	2-Oxoglutarate
M_glyald_c	D-Glyceraldehyde
M_fgam_c	N2-Formyl-N1-(5-phospho-D-ribosyl)glycinamide
M_dtmp_c	dTMP
M_adn_c	Adenosine

M_eig3p_c	D-erythro-1-(Imidazol-4-yl)glycerol 3-phosphate
M_25dhpp_c	2,5-Diamino-6-hydroxy-4-(5'-phosphoribosylamino)-pyrimidine
M_hpyr_c	Hydroxypyruvate
M_e4p_c	D-Erythrose 4-phosphate
M_3pg_c	3-Phospho-D-glycerate
M_orot_c	Orotate
M_peptido_syn_c	Peptidoglycan subunit of Synechocystis sp. PCC8603
M_gam6p_c	D-Glucosamine 6-phosphate
M_fum_c	Fumarate
M_for_c	Formate
M_ahcys_c	S-Adenosyl-L-homocysteine
M_4ahmmp_c	4-Amino-5-hydroxymethyl-2-methylpyrimidine
M_acg5sa_c	N-Acetyl-L-glutamate 5-semialdehyde
M_C04051_c	C04051
M_cbp_c	Carbamoyl phosphate
M_3mob_c	3-Methyl-2-oxobutanoate

Additional figures

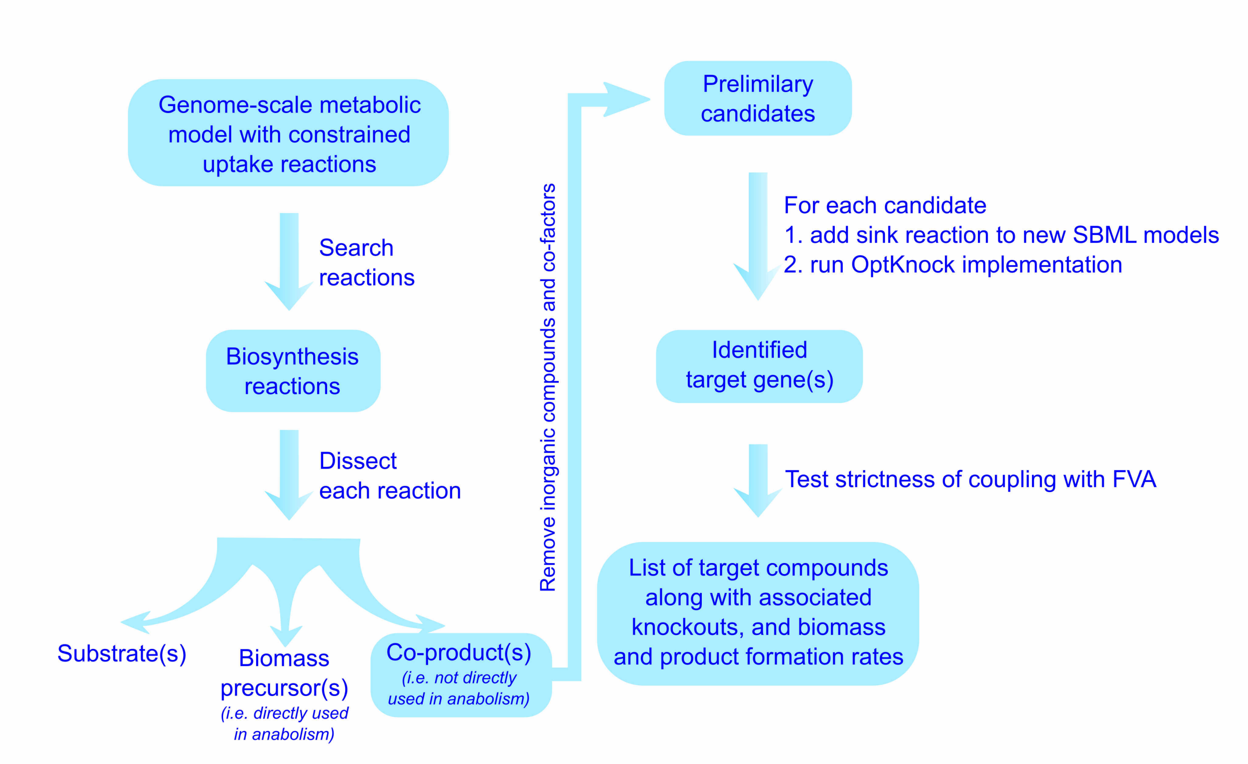

Figure S1. Graphical description of FRUITS.

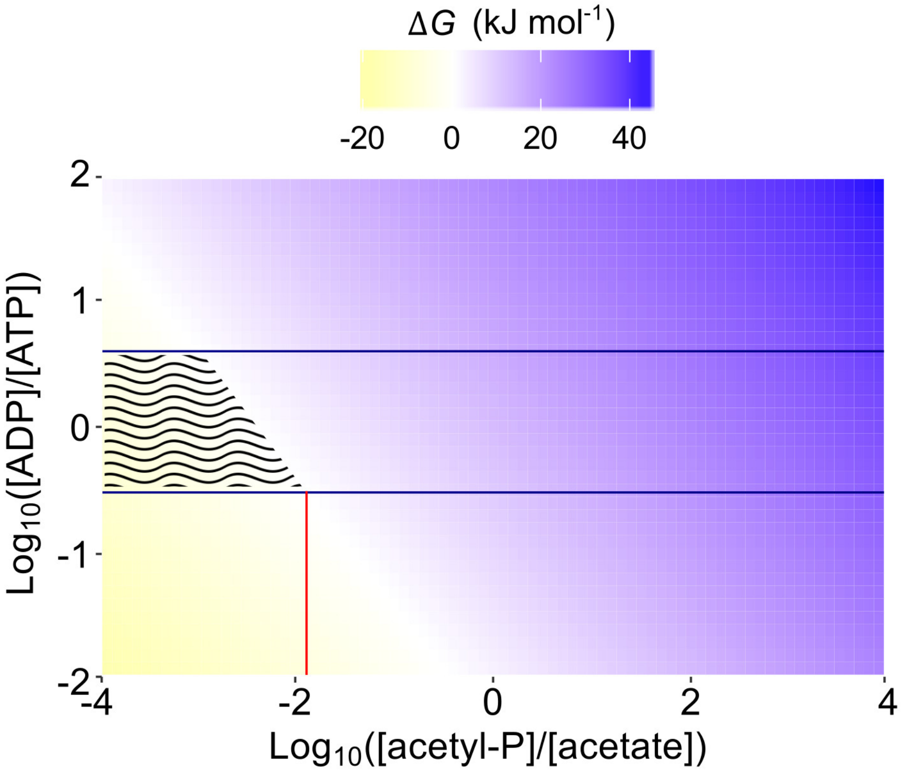

Figure S2. Thermodynamic analysis of the AckA pathway. Calculations are based on use of the eQuilibrator [1] for the reaction ATP + acetate ⇔ ADP + acetyl-P. The blue lines indicate the range of intracellular ADP:ATP ratio found for cyanobacteria [2]. The red line points to the maximal acetyl-P to acetate ratio, which would allow a negative ΔG within physiological conditions (area with the wavy pattern) of ADP:ATP reported for cyanobacteria.

References

1. Flamholz A, Noor E, Bar-Even A, Milo R. eQuilibrator--the biochemical thermodynamics calculator. Nucleic Acids Res. 2012;40 Database issue:D770-5.

2. Kallas T, Castenholz RW. Internal pH and ATP-ADP pools in the cyanobacterium Synechococcus sp. during exposure to growth-inhibiting low pH. J Bacteriol. 1982;149:229–36.
